# Bis(1*H*-imidazole-κ*N* ^3^)bis­(2-oxidopyridinium-3-carboxyl­ato-κ^2^ *O* ^2^,*O* ^3^)nickel(II)

**DOI:** 10.1107/S1600536809028347

**Published:** 2009-07-22

**Authors:** Bing-Yu Zhang, Jing-Jing Nie, Duan-Jun Xu

**Affiliations:** a Department of Chemistry, Zhejiang University, Hangzhou 310027, People’s Republic of China

## Abstract

In the crystal structure of the title Ni^II^ complex, [Ni(C_6_H_4_NO_3_)_2_(C_3_H_4_N_2_)_2_], the Ni^II^ atom is located on a twofold rotation axis and is chelated by two oxidopyridiniumcarboxyl­ate anions and further *cis*-coordinated by two imidazole ligands in a distorted *cis*-N_2_O_4_ octa­hedral geometry. The C—O bond distance of 1.2573 (19) Å found for the non-coordinating O atom of the carboxyl­ate group indicates significant delocalization of π-electron density over this residue. Similarly, the C—O bond distance of 1.260 (2) Å in the heteroaromatic ring indicates delocalization between the deprotonated hydr­oxy group and the pyridinium ring. The uncoordinated carboxyl­ate O atom links with the imidazole and pyridinium rings of adjacent mol­ecules *via* N—H⋯O and C—H⋯O hydrogen bonding, leading to a two-dimensional array parallel to (100).

## Related literature

For the nature of π-π stacking, see: Deisenhofer & Michel (1989 ▶); Xu *et al.* (2007 ▶); Li *et al.* (2005 ▶). For the short C—O bond distance between a pyridine ring and hydr­oxy-O atom in metal complexes of 2-oxidopyridinium-3-carboxyl­ate, see: Yao *et al.* (2004 ▶); Yan & Hu (2007*a* ▶,*b* ▶); Wen & Liu (2007 ▶).
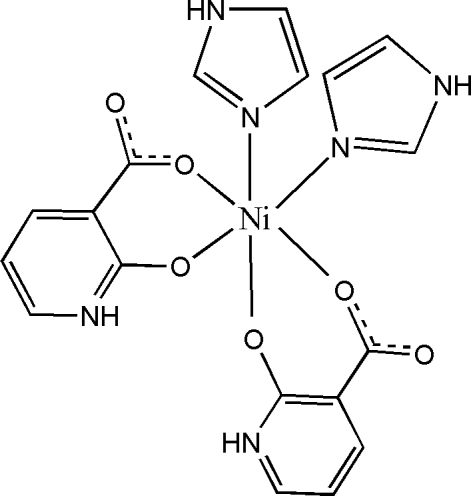


## Experimental

### 

#### Crystal data

[Ni(C_6_H_4_NO_3_)_2_(C_3_H_4_N_2_)_2_]
*M* *_r_* = 471.08
Monoclinic, 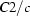

*a* = 16.5603 (12) Å
*b* = 9.9687 (7) Å
*c* = 12.7981 (9) Å
β = 111.203 (2)°
*V* = 1969.7 (2) Å^3^
*Z* = 4
Mo *K*α radiation
μ = 1.04 mm^−1^
*T* = 294 K
0.28 × 0.22 × 0.18 mm

#### Data collection

Rigaku R-AXIS RAPID IP diffractometer
Absorption correction: multi-scan (*ABSCOR*; Higashi, 1995 ▶) *T*_min_ = 0.730, *T*_max_ = 0.830
10787 measured reflections
1934 independent reflections
1690 reflections with *I* > 2σ(*I*)
*R*_int_ = 0.026

#### Refinement

*R*[*F* ^2^ > 2σ(*F* ^2^)] = 0.025
*wR*(*F* ^2^) = 0.067
*S* = 1.09
1934 reflections
141 parameters
H-atom parameters constrained
Δρ_max_ = 0.26 e Å^−3^
Δρ_min_ = −0.23 e Å^−3^

### 

Data collection: *PROCESS-AUTO* (Rigaku, 1998 ▶); cell refinement: *PROCESS-AUTO*; data reduction: *CrystalStructure* (Rigaku/MSC, 2002 ▶); program(s) used to solve structure: *SIR92* (Altomare *et al.*, 1993 ▶); program(s) used to refine structure: *SHELXL97* (Sheldrick, 2008 ▶); molecular graphics: *ORTEP-3* (Farrugia, 1997 ▶); software used to prepare material for publication: *WinGX* (Farrugia, 1999 ▶).

## Supplementary Material

Crystal structure: contains datablocks I, global. DOI: 10.1107/S1600536809028347/tk2499sup1.cif

Structure factors: contains datablocks I. DOI: 10.1107/S1600536809028347/tk2499Isup2.hkl

Additional supplementary materials:  crystallographic information; 3D view; checkCIF report

## Figures and Tables

**Table 1:** Hydrogen-bond geometry (Å, °)

*D*—H⋯*A*	*D*—H	H⋯*A*	*D*⋯*A*	*D*—H⋯*A*
N1—H1⋯O2^i^	0.86	1.93	2.7848 (19)	177
N3—H3⋯O2^ii^	0.86	2.03	2.796 (2)	148
C3—H3*A*⋯O3^iii^	0.93	2.41	3.323 (2)	167

Symmetry codes: (i) 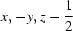; (ii) 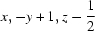; (iii) 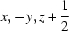.

**Fig. 1.**
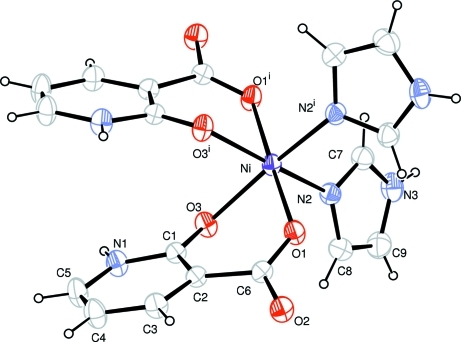
The molecular structure of (I) showing 40% probability displacement ellipsoids (arbitrary spheres for H atoms) [symmetry code: (i) 1 - x, y, 1/2 - z].

## supplementary crystallographic information

### Comment

As π-π stacking between aromatic rings is correlated with electron transfer process in some biological systems (Deisenhofer & Michel, 1989), metal complexes incorporating aromatic ligands have attracted much attention. As a part of an on-going investigation of π-π stacking (Xu *et al.*, 2007*a*, *b*; Li *et al.*, 2005), the title complex, (I), has been prepared and its crystal structure reported herein.

The analysis of (I) shows the Ni atom to be located on a 2-fold axis and to be chelated by two oxidopyridinium-carboxylate anions and two *cis*-orientated imidazole ligands to complete a distorted octahedral coordination geometry (Fig. 1). The carboxylate group is twisted with respect to the benzene ring with a dihedral angle of 22.09 (11)°. The C1—O3 bond distance of 1.260 (2) Å is much shorter than a normal single C—O bond, indicating delocalization of π-electron density over the deprotonated hydroxy group and the pyridinium ring, an observation which agrees with similiar features found in the other transition metal complexes of oxidopyridinium-carboxylate (Yao *et al.*, 2004; Yan & Hu, 2007*a*,b; Wen & Liu, 2007).

The uncoordinated carboxyl-O atom simutaneously links the imidazole and pyridinium rings *via* N—H···O hydrogen bonding leading to a 2-D array (Table 2). Weak C—H···O hydrogen bonding is also present in the crystal structure but no π-π stacking is evident.

### Experimental

2-Hydroxy-pyridine-3-carboxylic acid (0.13 g, 1 mmol), NaOH (0.04 g, 1 mmol), imidazole (0.14 g, 2 mmol) and NiCl_2_.6H_2_O (0.24 g, 1 mmol) were dissolved in water (15 ml). The solution was refluxed for 4.5 h. After cooling to room temperature, the solution was filtered. Single crystals of (I) were obtained from the filtrate after one week.

### Refinement

H atoms were placed in calculated positions with C—H = 0.93 and N—H = 0.86 Å, and refined in riding model approximation with *U*_iso_(H) = 1.2*U*_eq_(C,*N*).

### Crystal data

**Table:**

[Ni(C_6_H_4_NO_3_)_2_(C_3_H_4_N_2_)_2_]	*F*(000) = 968
*M**_r_* = 471.08	*D*_x_ = 1.589 Mg m^−^^3^
Monoclinic, *C*2/*c*	Mo *K*α radiation, λ = 0.71073 Å
Hall symbol: -C 2yc	Cell parameters from 3268 reflections
*a* = 16.5603 (12) Å	θ = 2.5–25.0°
*b* = 9.9687 (7) Å	µ = 1.04 mm^−^^1^
*c* = 12.7981 (9) Å	*T* = 294 K
β = 111.203 (2)°	Block, green
*V* = 1969.7 (2) Å^3^	0.28 × 0.22 × 0.18 mm
*Z* = 4	

### Data collection

**Table:**

Rigaku R-AXIS RAPID IP diffractometer	1934 independent reflections
Radiation source: fine-focus sealed tube	1690 reflections with *I* > 2σ(*I*)
graphite	*R*_int_ = 0.026
Detector resolution: 10.00 pixels mm^-1^	θ_max_ = 26.0°, θ_min_ = 2.4°
ω scans	*h* = −20→20
Absorption correction: multi-scan (*ABSCOR*; Higashi, 1995)	*k* = −11→12
*T*_min_ = 0.730, *T*_max_ = 0.830	*l* = −15→15
10787 measured reflections	

### Refinement

**Table:**

Refinement on *F*^2^	Primary atom site location: structure-invariant direct methods
Least-squares matrix: full	Secondary atom site location: difference Fourier map
*R*[*F*^2^ > 2σ(*F*^2^)] = 0.025	Hydrogen site location: inferred from neighbouring sites
*wR*(*F*^2^) = 0.067	H-atom parameters constrained
*S* = 1.09	*w* = 1/[σ^2^(*F*_o_^2^) + (0.0299*P*)^2^ + 1.5451*P*] where *P* = (*F*_o_^2^ + 2*F*_c_^2^)/3
1934 reflections	(Δ/σ)_max_ = 0.001
141 parameters	Δρ_max_ = 0.26 e Å^−^^3^
0 restraints	Δρ_min_ = −0.23 e Å^−^^3^

### Special details

**Table:**

Geometry. All e.s.d.'s (except the e.s.d. in the dihedral angle between two l.s. planes) are estimated using the full covariance matrix. The cell e.s.d.'s are taken into account individually in the estimation of e.s.d.'s in distances, angles and torsion angles; correlations between e.s.d.'s in cell parameters are only used when they are defined by crystal symmetry. An approximate (isotropic) treatment of cell e.s.d.'s is used for estimating e.s.d.'s involving l.s. planes.
Refinement. Refinement of *F*^2^ against ALL reflections. The weighted *R*-factor *wR* and goodness of fit *S* are based on *F*^2^, conventional *R*-factors *R* are based on *F*, with *F* set to zero for negative *F*^2^. The threshold expression of *F*^2^ > σ(*F*^2^) is used only for calculating *R*-factors(gt) *etc*. and is not relevant to the choice of reflections for refinement. *R*-factors based on *F*^2^ are statistically about twice as large as those based on *F*, and *R*- factors based on ALL data will be even larger.

### Fractional atomic coordinates and isotropic or equivalent isotropic displacement parameters (Å^2^)

**Table:**

	*x*	*y*	*z*	*U*_iso_*/*U*_eq_	
Ni	0.5000	0.24819 (3)	0.2500	0.02609 (11)	
N1	0.61183 (10)	−0.12600 (15)	0.30054 (12)	0.0340 (4)	
H1	0.6130	−0.1389	0.2346	0.041*	
N2	0.58315 (9)	0.39012 (14)	0.22850 (12)	0.0307 (3)	
N3	0.62487 (11)	0.55962 (16)	0.15138 (14)	0.0413 (4)	
H3	0.6224	0.6275	0.1088	0.050*	
O1	0.55981 (9)	0.25034 (11)	0.42031 (10)	0.0345 (3)	
O2	0.62229 (8)	0.17047 (12)	0.59099 (9)	0.0370 (3)	
O3	0.58766 (8)	0.09254 (12)	0.25646 (9)	0.0320 (3)	
C1	0.59865 (11)	0.00219 (17)	0.32922 (13)	0.0269 (4)	
C2	0.60022 (10)	0.01871 (17)	0.44182 (13)	0.0270 (4)	
C3	0.61083 (12)	−0.09161 (18)	0.50917 (14)	0.0347 (4)	
H3A	0.6108	−0.0806	0.5813	0.042*	
C4	0.62173 (15)	−0.22061 (19)	0.47285 (16)	0.0429 (5)	
H4	0.6278	−0.2947	0.5192	0.051*	
C5	0.62318 (15)	−0.23420 (18)	0.36860 (17)	0.0418 (5)	
H5	0.6320	−0.3184	0.3433	0.050*	
C6	0.59302 (11)	0.15609 (17)	0.48631 (13)	0.0273 (4)	
C7	0.55757 (13)	0.48832 (18)	0.15514 (15)	0.0362 (4)	
H7	0.5001	0.5059	0.1115	0.043*	
C8	0.67175 (12)	0.4006 (2)	0.27398 (16)	0.0404 (4)	
H8	0.7082	0.3442	0.3287	0.049*	
C9	0.69804 (13)	0.5053 (2)	0.22712 (18)	0.0456 (5)	
H9	0.7547	0.5343	0.2434	0.055*	

### Atomic displacement parameters (Å^2^)

**Table:**

	*U*^11^	*U*^22^	*U*^33^	*U*^12^	*U*^13^	*U*^23^
Ni	0.03550 (19)	0.02420 (17)	0.01909 (17)	0.000	0.01048 (13)	0.000
N1	0.0520 (9)	0.0317 (8)	0.0228 (7)	0.0040 (7)	0.0192 (7)	−0.0015 (6)
N2	0.0348 (8)	0.0290 (8)	0.0276 (7)	−0.0003 (6)	0.0106 (6)	0.0026 (6)
N3	0.0577 (11)	0.0309 (8)	0.0428 (9)	−0.0040 (7)	0.0272 (8)	0.0049 (7)
O1	0.0514 (8)	0.0281 (6)	0.0213 (6)	0.0057 (5)	0.0098 (6)	0.0002 (5)
O2	0.0551 (8)	0.0371 (7)	0.0175 (6)	0.0040 (6)	0.0117 (5)	−0.0025 (5)
O3	0.0475 (7)	0.0306 (6)	0.0229 (6)	0.0056 (5)	0.0190 (5)	0.0040 (5)
C1	0.0297 (8)	0.0290 (9)	0.0234 (8)	0.0004 (7)	0.0114 (7)	−0.0011 (7)
C2	0.0313 (9)	0.0301 (9)	0.0207 (8)	0.0008 (7)	0.0109 (7)	−0.0010 (7)
C3	0.0479 (11)	0.0357 (10)	0.0230 (9)	0.0020 (8)	0.0156 (8)	0.0016 (7)
C4	0.0682 (14)	0.0303 (10)	0.0339 (10)	0.0057 (9)	0.0229 (10)	0.0069 (8)
C5	0.0647 (14)	0.0268 (10)	0.0370 (11)	0.0057 (9)	0.0222 (10)	−0.0012 (8)
C6	0.0309 (9)	0.0317 (9)	0.0220 (8)	−0.0007 (7)	0.0127 (7)	−0.0021 (7)
C7	0.0415 (10)	0.0335 (10)	0.0335 (10)	−0.0003 (8)	0.0134 (8)	0.0034 (8)
C8	0.0371 (10)	0.0394 (11)	0.0419 (11)	0.0031 (8)	0.0108 (8)	0.0026 (8)
C9	0.0393 (11)	0.0432 (11)	0.0589 (13)	−0.0039 (9)	0.0233 (10)	−0.0059 (10)

### Geometric parameters (Å, °)

**Table:**

Ni—O1^i^	2.0422 (12)	O2—C6	1.2573 (19)
Ni—O1	2.0422 (12)	O3—C1	1.260 (2)
Ni—O3^i^	2.1059 (12)	C1—C2	1.441 (2)
Ni—O3	2.1058 (12)	C2—C3	1.369 (2)
Ni—N2	2.0610 (14)	C2—C6	1.504 (2)
Ni—N2^i^	2.0610 (14)	C3—C4	1.401 (3)
N1—C5	1.356 (2)	C3—H3A	0.9300
N1—C1	1.369 (2)	C4—C5	1.350 (3)
N1—H1	0.8600	C4—H4	0.9300
N2—C7	1.316 (2)	C5—H5	0.9300
N2—C8	1.373 (2)	C7—H7	0.9300
N3—C7	1.337 (2)	C8—C9	1.352 (3)
N3—C9	1.361 (3)	C8—H8	0.9300
N3—H3	0.8600	C9—H9	0.9300
O1—C6	1.250 (2)		
			
O1^i^—Ni—O1	178.80 (6)	O3—C1—C2	127.05 (15)
O1^i^—Ni—N2	86.55 (5)	N1—C1—C2	115.33 (14)
O1—Ni—N2	92.62 (5)	C3—C2—C1	119.31 (15)
O1^i^—Ni—N2^i^	92.62 (5)	C3—C2—C6	120.19 (14)
O1—Ni—N2^i^	86.55 (5)	C1—C2—C6	120.47 (14)
N2—Ni—N2^i^	93.29 (8)	C2—C3—C4	122.08 (16)
O1^i^—Ni—O3^i^	84.52 (5)	C2—C3—H3A	119.0
O1—Ni—O3^i^	96.37 (5)	C4—C3—H3A	119.0
N2—Ni—O3^i^	170.03 (5)	C5—C4—C3	118.07 (17)
N2^i^—Ni—O3^i^	91.53 (5)	C5—C4—H4	121.0
O1^i^—Ni—O3	96.37 (5)	C3—C4—H4	121.0
O1—Ni—O3	84.52 (5)	C4—C5—N1	120.47 (17)
N2—Ni—O3	91.53 (5)	C4—C5—H5	119.8
N2^i^—Ni—O3	170.03 (5)	N1—C5—H5	119.8
O3^i^—Ni—O3	85.08 (7)	O1—C6—O2	122.70 (15)
C5—N1—C1	124.68 (15)	O1—C6—C2	120.28 (14)
C5—N1—H1	117.7	O2—C6—C2	117.02 (15)
C1—N1—H1	117.7	N2—C7—N3	111.32 (17)
C7—N2—C8	105.36 (15)	N2—C7—H7	124.3
C7—N2—Ni	123.21 (12)	N3—C7—H7	124.3
C8—N2—Ni	131.20 (12)	C9—C8—N2	109.68 (17)
C7—N3—C9	107.57 (16)	C9—C8—H8	125.2
C7—N3—H3	126.2	N2—C8—H8	125.2
C9—N3—H3	126.2	C8—C9—N3	106.06 (17)
C6—O1—Ni	129.67 (11)	C8—C9—H9	127.0
C1—O3—Ni	117.96 (10)	N3—C9—H9	127.0
O3—C1—N1	117.62 (14)		

Symmetry codes: (i) −*x*+1, *y*, −*z*+1/2.

### Hydrogen-bond geometry (Å, °)

**Table:**

*D*—H···*A*	*D*—H	H···*A*	*D*···*A*	*D*—H···*A*
N1—H1···O2^ii^	0.86	1.93	2.7848 (19)	177
N3—H3···O2^iii^	0.86	2.03	2.796 (2)	148
C3—H3A···O3^iv^	0.93	2.41	3.323 (2)	167

Symmetry codes: (ii) *x*, −*y*, *z*−1/2; (iii) *x*, −*y*+1, *z*−1/2; (iv) *x*, −*y*, *z*+1/2.
